# On a Simple Method of Ascertaining, without the Use of the Catheter Whether the Eustachian Tubes Are Pervious; with Some Observations on the Treatment of Cases of Obstruction in These Tubes

**Published:** 1853-05

**Authors:** Joseph Toynbee


### SURGERY.

*On a simple Method of ascertaining, without the use of the Catheter whether the Eustachian Tubes are pervious; with some observations on the Treatment of cases of Obstruction in these Tubes.* By JOSEPH TOYNBEE, F. R. S.—The author pointed out the objections to the two ordinary modes of exploring the Eustachian tubes—viz. that the use of the catheter is liable to produce pain and discomfort; that, without experience, it is not easy to ascertain whether it be really in the tube; that the plan of attempting to distend the tympanum by a forcible expiration, while the mouth and nostrils are kept closed, is not always successful, from the fact that the young and nervous cannot be taught to perform the act, and that sometimes, when it is properly done, the guttural orifices of the tubes seem to be pressed together so as to preclude the air from entering. In a paper recently read before the Royal Society, the author endeavored to show that the guttural orifice of each Eustachian tube is generally closed, and that the air in the tympanum is not continuous with that in the cavity of the fauces, except during the momentary act of deglutition. In proof of this the following experiment was cited: If the mouth be shut, and the nostrils be held closed by the finger and thumb, and then the act of swallowing be performed, a sensation of fulness or pressure is experienced in each ear; and this sensation does not disappear upon the removal of the pressure from the nose, but it vanishes at once when the act of swallowing is again performed, while the mouth and nostrils are open. During the first act of swallowing, a small quantity of air was forced into the tympanitic cavities through the Eustachian tubes, and it therein remained until the second act of swallowing again opened the tubes and permitted the air to escape. The muscles whereby the Eustachian tubes are opened are the tensor and levator palati, which it is well known take origins from the cartilaginous walls of the tubes. As, during the act of swallowing with closed mouth and nostrils, air is forced through the Eustachian tubes into the tympanitic cavities, it is evident that the permeability of these tubes can be ascertained by making the patient swallow ssme saliva while the mouth and nose are shut. Nor need the surgeon depend upon the statement of the patient respecting the sensation of distension felt in the ears; for, by listening with the *otoscope*, should the Eustachian tubes be pervious, the air will be distinctly heard to enter the tympanitic cavities, and produce a gentle crackling sound. The author next proceeded to consider the treatment of cases of obstruction of the Eustachian tubes, especially in reference to the use of the catheter. It having been ascertained that these tubes are obstructed, is it desirable to attempt to open them by means of the catheter? Believing that obstruction in the Eustachian tubes generally depends upon a thickened state of the mucous membrane covering the guttural orifice, and that this state is always associated with a thickened condition of the faucial mucous membrane and of the mucous membrane of the tympanum, the author suggests—especially to those inexperienced in the use of the catheter, not to attempt to pass this instrument—firstly, because, in such cases, the mucous membrane of the Eustachian tube is often so tumefied that no ordinary degree of pressure will force the air into the tympanum; and, secondly, because, should the surgeon succeed in transmitting a few air-bubbles, the relief obtained is only partial and endures for a very brief period, since the mucous membrane remains as thick as before, and the ill effects of the obstruction soon recur, from the air in the tympanum becoming of a different density from that without. The membrana tympani becomes more or less fixed. The treatment recommended is such as shall tend to reduce the thickened mucous membrane of the guttural orifices of the Eustachian tubes to a healthy size, so that their muscles may be able to open them. For this purpose, besides the use of general remedies, the solid nitrate of silver, or a strong solution of hydrochloric acid, may be applied to the mucous membrane of the fauces and to the apertures of the tubes, and gentle counter-irritation is to be kept up over the region of the fauces. By these measures, as a general rule, the mucous membrane can be reduced to its natural state, and the tubes become again opened by their muscles. Should this not take place, the Eustachian catheter may now and then be introduced, and air be gently blown through it. A modification in the shape of the Eustachian catheter is suggested—viz. that it should be oval instead of round, the advantages derived being, that it not only can be passed through the nose with less discomfort to the patient, but its presence in the Eustachian tube is much less disagreeable from the absence of the convex surfaces which, in the rounded catheter, press against the nearly flat surfaces of the tube. In conclusion, the author expresses his concurrence in the opinion of Harvey and Kramer, that enlarged tonsils are never the cause of obstruction in the Eustachian tubes, and that any benefit that may have followed their extirpation has arisen from the loss of blood consequent upon the operation.—*Lancet.*

